# *peaksat*: an R package for ChIP-seq peak saturation analysis

**DOI:** 10.1186/s12864-023-09109-7

**Published:** 2023-01-25

**Authors:** Joseph R Boyd, Cong Gao, Kathleen Quinn, Andrew Fritz, Janet Stein, Gary Stein, Karen Glass, Seth Frietze

**Affiliations:** 1 Department of Biomedical and Health Sciences, University of Vermont, 106 Carrigan Drive, 302 Rowell, VT 04505 Burlington, USA; 2 Department of Biochemistry, University of Vermont, Burlington, VT USA; 3 University of Vermont Cancer Center, Burlington, VT USA; 4 Department of Surgery, University of Vermont, Burlington, VT USA; 5 Department of Pharmacology, University of Vermont, Burlington, VT USA

**Keywords:** Peak saturation, Read depth estimate, ChIP-Seq

## Abstract

**Background:**

Epigenomic profiling assays such as ChIP-seq have been widely used to map the genome-wide enrichment profiles of chromatin-associated proteins and posttranslational histone modifications. Sequencing depth is a key parameter in experimental design and quality control. However, due to variable sequencing depth requirements across experimental conditions, it can be challenging to determine optimal sequencing depth, particularly for projects involving multiple targets or cell types.

**Results:**

We developed the *peaksat* R package to provide target read depth estimates for epigenomic experiments based on the analysis of peak saturation curves. We applied *peaksat* to establish the distinctive read depth requirements for ChIP-seq studies of histone modifications in different cell lines. Using *peaksat,* we were able to estimate the target read depth required per library to obtain high-quality peak calls for downstream analysis. In addition, *peaksat* was applied to other sequence-enrichment methods including CUT&RUN and ATAC-seq.

**Conclusion:**

*peaksat* addresses a need for researchers to make informed decisions about whether their sequencing data has been generated to an adequate depth and subsequently sufficient meaningful peaks, and failing that, how many more reads would be required per library. *peaksat* is applicable to other sequence-based methods that include calling peaks in their analysis.

**Supplementary Information:**

The online version contains supplementary material available at 10.1186/s12864-023-09109-7.

## Introduction

Recent advances in epigenetic profiling technologies have revolutionized our understanding of the regulatory architecture of the genome. Global enrichment profiling techniques such as chromatin immunoprecipitation combined with high-throughput sequencing (ChIP-seq) and its derivates are widely used techniques for mapping the genome-wide binding sites of chromatin-associated proteins, including transcription factors (TFs), and histone posttranslational modifications (PTMs) [1–3]. Together with other comprehensive functional data such as transcriptomic (RNA-seq), chromatin accessibility (ATAC-seq) and higher-order chromatin interaction data (i.e. Hi-C or ChIA-PET), provide the information necessary for constructing cell type-specific reference epigenomes, and provide valuable information that supports emerging analyses of genome structure and function (refs reviewed in [4–8]).

A critical quality control consideration, both in the design and data quality assessment for ChIP-seq experiments, is to understand the sequencing depth required to obtain a high-quality dataset. Adequate sequencing depth depends on the target factors, size of the genome, as well as the number and size of the binding sites of the protein [9]. Useful guidelines have been provided by the modENCODE and ENCODE consortia about the required read depth to obtain adequate high-quality data for downstream analysis [10–12]. Despite the recommendations, sequencing libraries can be of low-quality due to technical problems, displaying weak or no enrichment compared to input or negative control. In that case, increased sequencing will not add to the quality of the dataset and it will be necessary to repeat the experiment. Therefore, the development of effective, easy-to-use tools for informing decisions on whether further sequencing with increased read depth would improve data quality would be broadly beneficial to the field.

Here, we introduce *peaksat*, a new tool developed to evaluate read requirements and peak saturation in ChIP-seq experiments. *peaksat* can be applied more generally for estimating target read depths for other sequence-enrichment studies, including CUT&RUN and ATAC-seq. *peaksat* is an open-source R package with minimal dependencies. Here, the usability of *peaksat* is demonstrated with a case study that investigates the distinctive read depth requirements for ChIP-seq analysis of two closely related histone H4 acetylation PTMs across different human breast cancer cell lines.

## Materials & methods

### Cell culture & ChIP-seq

Cell lines were cultured as described previously in [13]. The human breast cancer cell line MCF10A [14] was purchased from the ATCC, and the cell lines MCF10AT1 [15], MCF10CA1a [16] and MCF10DCIS [17] are a gift from Jeff Nickerson’s lab. ChIP-seq assays for histone H4 H4K5ac and H4K8ac were performed as described by O’Green et al. [18]. Biological replicates for ChIP-seq experiments were generated for each cell line from different culture passages. Input libraries were used as the ChIP controls. Each library was sequenced across two separate lanes of 75 bp single end Illumina sequencing. Since histone H4 acetylation modifications are not well characterized in these cell lines, we performed the initial sequencing with an arbitrary depth of 10-15 M reads. We then further sequenced these samples with re-pooled libraries to reach the read depth estimated by *peaksat* required to reach peak saturation. Raw reads were aligned to the human genome (hg38) using STAR aligner (version 2.7.7a) using the parameter --alignIntronMax 1 to disable spliced alignments [19]. Data quality was assessed using the *seqsetvis* [20] and *ssvQC* R package (version 1.0.6) [21] using 1000 peaks randomly selected from the merger of all peak calls per histone modification in terms of replicate overlap and FRIP (fraction of reads in peaks, [10]).

### Peak saturation pipeline and quality control analysis

We developed the *peaksat* R package to estimate target read depth required for epigenomic profiles generated by ChIP-seq and other sequence-based enrichment assays. The required input files are bam files generated by any aligner used to analyze ChIP-seq data. If the number of total expected peaks is not known, i.e., for an uncharacterized factor, then the creation of a meta-pool that includes all libraries available for this factor is recommended. The purpose of the meta-pool is to include enough reads in a single sample so that saturation will be reached with at least the meta-pool, if that is possible with the data provided. The *peaksat* pipeline is capable of organizing data by ChIP-seq target factor and then creating pooled bam files for any biological replicates. Once the aligned bam files have been assembled, then the primary processing step of *peaksat* can be applied to unpooled replicates, pooled replicates, and meta-pools.

**Fig. 1:**
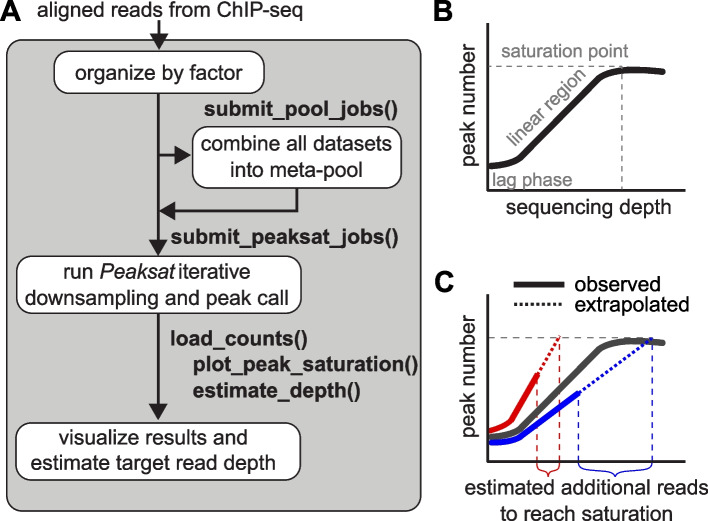
*peaksat* package to evaluate read depth in ChIP-seq experiments. **A** The *peaksat* workflow is shown here. ChIP-seq projects are organized by the factor (i.e., target TF or histone mark), and then all available data for a factor are combined into a meta-pool for a specific target. **B** The typical relationship of number of called peaks vs sequencing depth. **C** The red and blue lines indicate saturation curves for the different libraries composing the meta-pool. Solid lines represent observed peak count vs read count data while the dotted lines are extrapolations of those trends

Then *peaksat* (version 1.0.0; R version 4.1.2; *Bioconductor* release version 3.14) is used to coordinate down sampling (samtools view -s, version 1.3.1) and subsequent peak calling. Narrow peaks were called against input using MACS2 callpeak (version 2.1.1.20160309) [19], with the default q-value threshold of 0.01 by MACS2. We used FE (fold-enrichment) cutoff of 1 in the initial sequencing to estimate the additional read depth required, and then we used FE cutoff of 5 in the combined sequencing to explore the peak saturation profile. The main analytical steps of the *peaksat* workflow are summarized in Fig. 1.

For each ChIP-seq pooled or unpooled library, *peaksat* performs iterative down sampling of reads and subsequent peak calling to establish a curve of peak number vs read count (Fig. 1B). If the sequencing depth has reached a saturation point, the plateau shown in Fig. 1B, it is no longer cost-effective to sequence deeper in order to gain more meaningful peaks. If this curve represents a meta-pool, we can use the number of peaks at saturation as a target number of peaks when evaluating the un-characterized histone modification target.

Between an initial lag phase and saturation, observed peak saturation curves normally show a linear relationship between peak count and read depth (Fig. 1B&C). Therefore, in this part, *peaksat* fits a linear regression model and extrapolates to the target number of peaks (either derived from the meta-pool or manually specified) to estimate the number of new reads required for the specific target to reach saturation (Fig. 1C). The libraries composing the meta-pool are expected to saturate at the same number of target peaks (saturation point of meta-pool), but some libraries exhibit a shallower slope and therefore require deeper sequencing to reach the same point. The read count where the extrapolated trends intersect with the target number of saturated peaks is the target read count to reach saturation.

### Software implementation

*peaksat* is an R package (https://www.r-project.org/) for the evaluation of ChIP-seq and other sequence-enrichment assays (see code availability section for full details). The package provides functions to support two primary tasks: 1) iteratively subsampling aligned bam files and calling MACS2 callpeak, and 2) analyzing and visualizing the resulting peak count according to the down- sampled read depth. Task #1 is highly computationally intensive and so *peaksat* leverages the distributed computing capabilities provided by either the SGE or SLURM job schedulers. If those are unavailable, *peaksat* will utilize the *parallel* R package to speed up processing locally. Processing outputs are organized into a directory structure; previously completed sub-tasks will not be rerun by default so partial processing runs can be readily resumed. Task #2 provides regression functions to predict peak saturation points as more reads are added and various visualizations to aid in analysis.

### CUT&RUN & ATAC-seq data

CUT&RUN data were downloaded for GSE172130 [21]. Reads were aligned to the mm10 reference genome using bowtie2 (version 2.2.9) following the CUT&RUNTools processing pipeline [22]. Peaks were called using MACS2 as part of *peaksat* using pooled IgG samples as input and with --format BAMPE [19].

ATAC-seq data were downloaded for GSE161501 [23]. Reads were aligned to the hg38 reference genome using bowtie2 (version 2.2.9) as part of pipeline for ATAC-seq analysis [24, 25]. Otherwise, processing steps was the same as described for ChIP-seq with the exception that no input sample was used for calling peaks.

## Results and discussion

### *peaksat* pipeline facilitated profiling of histone H4 acetylation patterns in a breast cancer progression cell model

We applied *peaksat* to study the histone H4 Lys5 and Lys8 acetylation (H4K5ac and H4K8ac) ChIP-seq patterns in the MCF10A human breast cancer progression model consisting of four distinct cell lines: the normal-like epithelial MCF10A cells [14], premalignant MCF10AT1 cells [15] that express constitutively activated HRAS [15], and the subsequent xenograft derivatives, MCF10DCIS (ductal carcinoma in situ) cells [17] and MCF10CA1a metastatic cells [16]. We generated replicate ChIP-seq datasets for H4K5ac and H4K8ac in each cell lines conducted a pilot round sequencing at a relatively shallow sequencing depth and obtained an average of 11 million reads with no more than 5 k peaks per individual dataset (Fig. S1A&B, Fig. S2A&B). The FRIP (Fraction of Reads in Peak) values indicated these libraries have low background signal (Fig. S1C and S2C), and replicate analysis showed overall concordance for replicates for each target in different cells (Fig. S1D-E and S2D-E). Initial quality control analysis showed this was insufficient depth for a robust peak call but indicated that the ChIP-seq libraries had been successful for both marks (Supplemental figs. S1 and S2). In addition, we found each H4Kac mark shared highly similar patterns of peak enrichment across different cell lines, despite low frequency of peak overlaps between biological replicates (S1D-G and S2D-G).

**Fig. 2:**
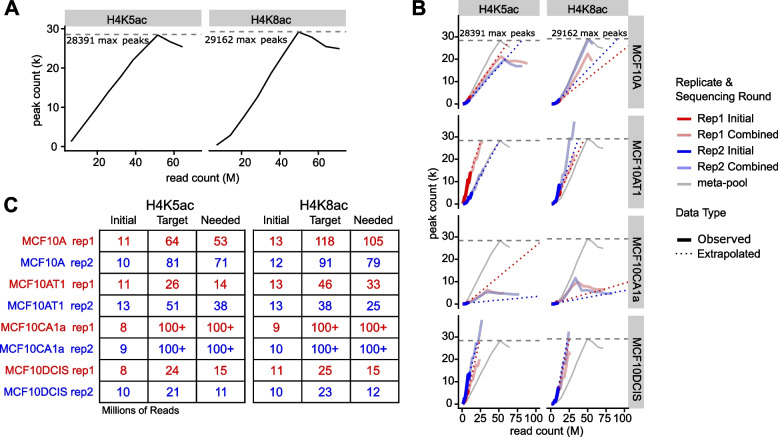
*Peaksat* package estimates sequence depth required to reach saturation for ChIP-seq. **A** Meta-pool saturation curves that show the number of peaks (thousands, k) versus the number of reads (millions, M) for the meta-pool of H4K5ac and H4K8ac ChIP-seq libraries. **B** Target depth estimation procedure. Blue and red lines indicate data from constituent replicates to the meta-pool. Solid-dark lines are observed data from the initial low-depth sequencing run. Dotted lines are linearly extrapolated from the initial sequencing and were used to predict the number of reads required to reach saturation. Solid-light lines are observed data from combined initial and follow-up sequencing runs. **C** Tables used to calculate the number of additional reads required to reach saturation (Needed) based on the number of reads initially sequenced (Initial) and the estimated total target reads to reach peak saturation (Target). All values are in millions of reads

To determine how much more sequencing was required to have enough read depth to support a rigorous analysis, we combined all replicates per histone modification into meta-pools aiming to approximate the saturated peak count for each target. The results estimated 51.5 million reads for H4K5ac and 49.7 million reads for H4K8ac were sufficient to reach saturation, hitting maximum peak counts of 28,400 and 29,200, respectively **(**Fig. 2A). These maximum number of peaks for each meta-pool are representative of the peak saturation point beyond which additional sequencing is not necessary. Consistent with the previous observations for other targets, we found the majority of each peak saturation curve appeared to be linear where increases in peak count were directly proportional to increases in read count. Additionally, we investigated the suitability of a linear regression model to describe the linear portion of the peak saturation curve. The resulting linear models fit the saturation profiles with an adjusted R^2^ value around 90% (Table S1). In contrast to the meta-pools, none of the individual libraries appeared to approach peak saturation, with peak counts ranging from 420 to 13,690 (Fig. 2B&C).

To determine how many additional reads were needed to reach peak saturation, we used the corresponding meta-pool saturation peak count and extrapolated a linear regression to obtain a read depth estimate per library (Fig. 2B&C). Based on these estimates, we performed a second round of sequencing aimed at obtaining enough read depth for each sample. As expected, the combined datasets exhibited marked improvement in the number of total peaks and an increased concordance of peaks between replicates and across cell lines (Fig. S3 and Fig. S4). Interestingly, the peak saturation profiles were inconsistent across cell lines for a single mark, and this variability was more pronounced in the H4K5ac peak enrichment profiles (Fig. 2B). The MCF10A H4K5ac replicates demonstrated sufficient read depth and reached peak saturation earlier than that estimate of the meta-pool, however, they did not show high enrichment compared to the meta-pool peak enrichment profile. In contrast, the MCF10AT1 and MCF10CA1a replicates for H4K5ac were expected to reach saturation around the meta-pool peak saturation point, but neither replicate was saturated, suggesting a need for additional sequencing to improve the dataset and increase the number of meaningful peaks. The MCF10DCIS cell line also showed a sharp increase in H4K5ac peak number with additional read depth. For H4K8ac, the saturation curves showed similar cell line specific patterns as that of H4K5ac. The discrepancy in read requirements for the same targets across different cell lines suggest that peak saturation and read requirements could be due to underlying heterogeneity across these cell lines.

### Additional factors influencing peak saturation

**Fig. 3:**
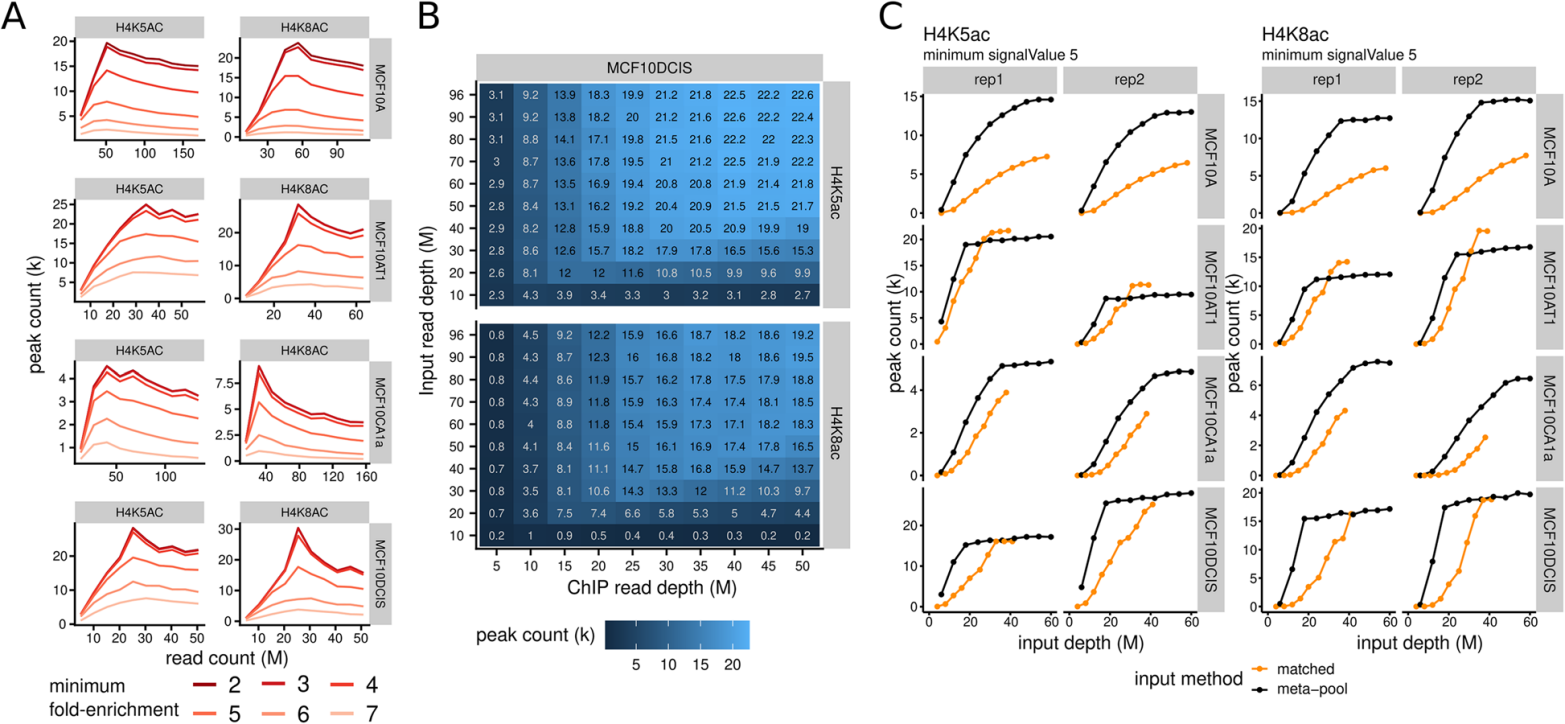
The impacts of fold-enrichment threshold and input depth on peak saturation. **A** Number of peaks versus the number of reads across different fold-enrichment thresholds (mapped to shades of red). Data shown are from the pooled replicates with combined sequencing rounds. **B** Heatmap of the number of peaks called for combinations of input and ChIP read depths. Numbers along with the color scale indicate thousands of peaks. **C** Number of peaks versus input read depth curves for all cell lines. All sequencing depths have been limited to 50 M reads for this analysis

We used *peaksat* to investigate the influence of fold-enrichment and input control read depth on peak saturation. Increasing the minimum fold-enrichment values for peak analysis (see methods) showed an expected decrease in the saturation peak number (Fig. 3A). Notably, when the minimum FE threshold is set at a value greater than 5, the peak enrichment never reaches saturation. MACS2 calls peaks against input utilizing dynamic Poisson distribution to effectively capture the local enriched peaks against those in input [19]. This peak calling algorithm and the read depth of input control in ChIP-Seq (as suggested by ENCODE) are both important factors involved in peak saturation [10, 19]. Through comparison of the peak saturation patterns under different down-sampling of input reads, we investigated whether increasing the number of control input reads enable saturation. For both targets, H4K5ac and H4K8ac, peak numbers saturate at 50–60 million input reads (Fig. 3B). Given sufficient input reads, the incremental target read depth drives peak enrichment to saturate and before plateauing (Fig. 3C). Taken together, both fold-enrichment threshold and sufficient read depth of input controls will help in estimating saturated read depth for targets and achieving high quality data for downstream analysis.

### H4K5ac and H4K8ac share similar binding profiles

**Fig. 4:**
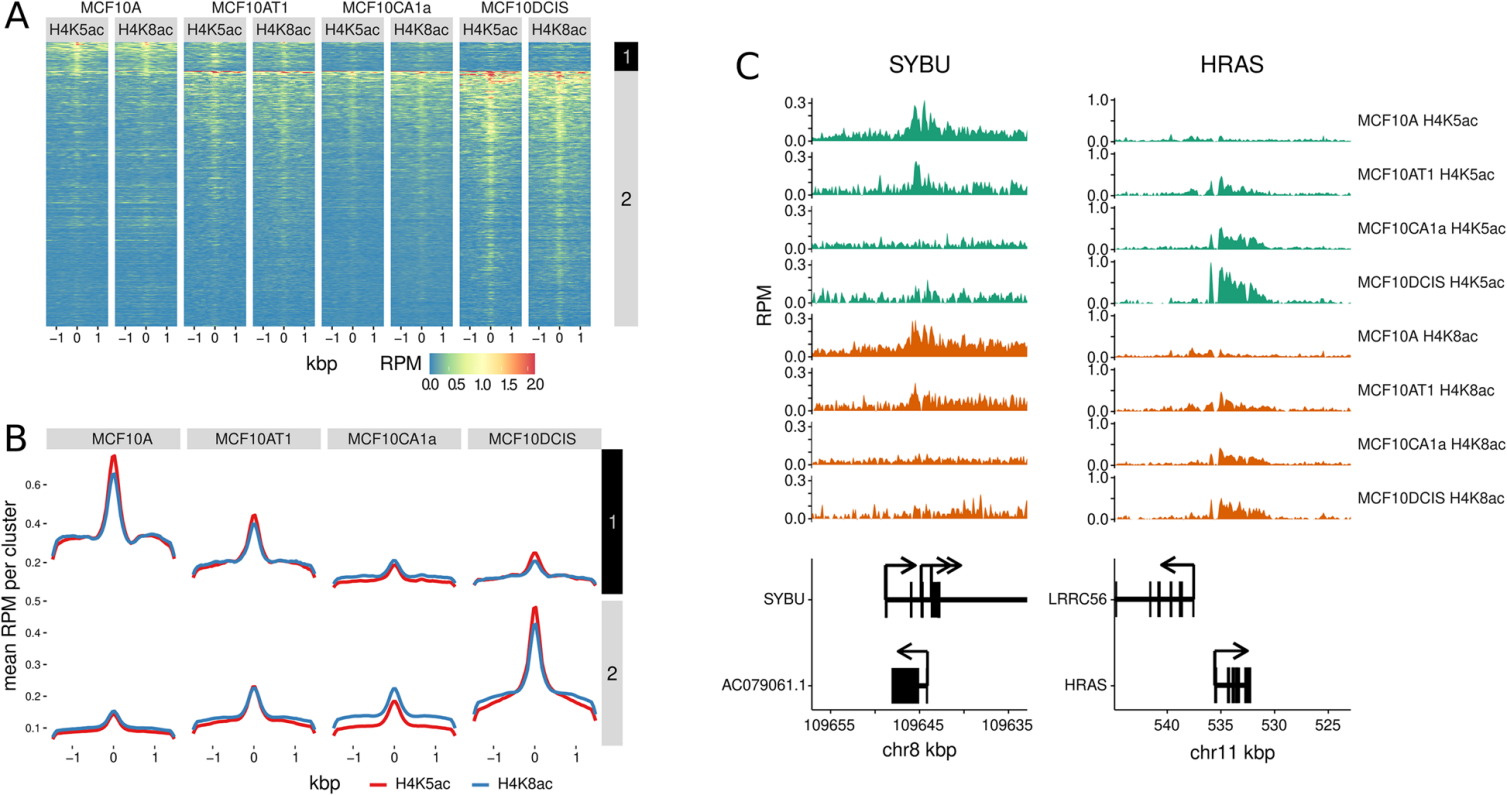
Similar binding profiles between H4K5ac and H4K8ac. **A** Differential binding peaks across cell lines for H4K5ac and H4K8ac are combined together and grouped into 2 clusters to show the altered binding profiles and peak enrichment during progression cell models. The y-axis shows the different peaks and x-axis show the sites around the peak summit. RPM, reads per million, is scaled to indicate the enrichment signal. **B** Overall enrichment profiles of differential binding sites centered at peak summit for both targets across different cell lines. The clusters are the same as that in Fig. 4A. **C** Examples of genes nearest to the peaks in cluster 1 and cluster 2. Green peaks refer to H4K5ac binding sites, and orange peaks refer to H4K8ac binding sites. The arrows indicate the transcription direction (strand) for each example gene

Overall, H4K5ac and H4K8ac showed similar enrichment profiles across the breast cancer progression cell lines (Fig. 4A&B). To compare binding patterns for these targets, we performed pairwise differential enrichment analysis of each mark across cell lines and identified significantly differentially enriched peaks that could be grouped into two clusters based on enrichment signal. Cluster 1 were H4K5ac and H4K8ac peaks more enriched in MCF10A cells, whereas Cluster 2 were peaks more enriched in the tumorigenic derivative cell lines (MCF10A-T1, −DCIS and -CA1a) compared to MCF10A. For example, the enrichment of H4K5ac and H4K8ac near the *SYBU* transcription start site (TSS) show a similar pattern of enrichment profiles that are stronger in normal MCF10A cell than the tumorigenic derivative cell lines (Fig. 4C). Conversely, H4K5ac and H4K8ac was more strongly enriched near the *HRAS* TSS in tumorigenic cells MCF10AT1, MCF10DCIS, MCF10CA1a compared to MCF10A cells. These results indicate overall similarity between H4K5ac and H4K8ac profiles that may change with increased proliferative capacity of the cells. Thus, *peaksat* provided an appropriate sequencing depth estimate required to identify differential histone H4 acetylation modifications across different cell lines.

### *Peaksat* pipeline is generally applicable for peak enrichment analysis with other assays

We further applied *peaksat* to evaluate the peak saturation curves of ChIP-seq data for transcription factors (TFs), as well as CUT&RUN and ATAC-seq datasets in order to evaluate its general applicability. We evaluated previously published ChIP-seq data sets that compared CTCF profiles in the MCF10A progression model [13]. These results show the read depth required to reach peak saturation for individual replicates across 3 different cell lines (Fig. S5A). We also evaluated peaksat for quality control of different cell preparation methods for CUT&RUN analysis of H3K4me3 and Ikaros, a zinc finger transcription factor [11]. Regardless of sample preparation (frozen or fresh), the PTM H3K4me3 required only 10 M reads to reach saturation, and a linear regression relationship exists between read depth and peak count before saturation (Fig. S5B). In contrast, the Ikaros TF saturation profiles were variable across the different preparations. ATAC-seq peaks were evaluated from different datasets generated from different leukemia subtypes [16] showing that 100 M reads fit well with linear model based (Fig. S5C). These results together suggest that the *peaksat* linear regression model is useful to estimate the saturation and target read depth estimates for other sequence- and peak-based assays.

### *Peaksat* performance

*peaksat* is reasonably fast, particularly for datasets with a few samples or shallow sequencing depth. *peaksat* has primarily been tested using SGE (Sun Grid Engine) for job scheduling. It also has support for SLURM and even bash processing if neither job scheduler is available. Typical runs for peaksat are approximately 1 hour.

## Discussion

Read depth has a major impact on the downstream analysis on high-throughput sequencing enrichment assays such as ChIP-seq [11]. In practice, it is important to be able to predict the number of sequencing reads required to saturate the detection of peaks. However, no R packages currently exist that flexibly evaluates peak saturation and read depth. We therefore developed *peaksat* to provide a reliable estimate of sequencing depth as an important quality control measure and tool for ChIP-seq pipelines. By combining all data into a meta-pool and sampling stepwise fractions of the reads, *peaksat* determines peak saturation and compares these predictions with the set of total peaks identified from the complete data. In practical terms, a lack of saturation point is important for the study design and suggests that it would be difficult to define an appropriate sequencing depth and that other criteria must be specified.

We applied *peaksat* to a ChIP-seq case study and investigated the impact of sequencing depth in multiple H4K5ac and H4K8ac ChIP-seq experiments using MCF10A progression model consisting of four different cell lines. For each mark, all reads were combined from both replicates to form a meta-pool of all reads to reach saturation. We then compared datasets for individual histone marks with their read-subsampled datasets. Importantly, our results showed that the relationship between saturated peaks and the number of sequenced reads may be extrapolated to estimate the sequencing depth requirement for ChIP-seq experiments. Notably, this analysis revealed that H4K5ac and H4K8ac modifications have different read depth requirements across different cell lines. As illustrated in Fig. 2B, the linear models for both histone PTMs in MCF10DCIS seem never to saturate, approaching 60 k peaks, a preposterous number considering the expected number of peaks based on the meta-pool saturation peak count. While these additional peaks could reflect genuine sites of H4K5/K8 acetylation, it is possible that these peaks instead reflect histone H4 acetylation PTMs present only in a small fraction of cells or sites which undergo particularly transient and labile acetylation states. Consequently, determining whether these peaks should be removed due to the uncertain biological meaning, and whether they will affect peak saturation profile presented a challenge. This is consistent with previous studies have demonstrated that high quality ChIP-Seq data never saturates without a fold-enrichment cutoff [19]. Overall, *peaksat* enabled us to reach target read depth estimates in order to perform downstream differential enrichment analysis. This revealed an overall similar binding profiles between H4K5ac and H4K8ac marks, but also showed distinct patterns between normal and tumorigenic breast cell lines.

The primary utility of *peaksat* is to provide an important starting point for ChIP-seq studies to determine if the sequence data generated is sufficient to perform downstream analysis. Indeed, as sequencing depth increases, the number of false positive peaks may increase and it is important to use guidelines provided by ENCODE to evaluate false peaks in ChIP-seq data. Thus, a particularly important downstream analysis is identifying a set high-confident peak sets from replicates.

## Conclusions

The *peaksat* R package is useful in conducting and evaluating ChIP-seq projects. We expect it to easily integrate into existing pipelines as it leverages well established and commonly used bioinformatics utilities: MACS2, samtools, and R. *peaksat* is a useful tool to apply into ChIP-seq, CUT&RUN, ATAC-seq, and other high-throughput sequencing assays to facilitate high-quality data and a deeper biological understanding.

## Supplementary Information

**Additional file 1: Figure S1.** QC analysis of H4K5ac initial sequencing.

**Additional file 2: Figure S2.** QC analysis of H4K8ac initial sequencing.

**Additional file 3: Figure S3.** QC analysis of H4K5ac combined sequencing.

**Additional file 4: Figure S4.** QC analysis of H4K8ac combined sequencing.

**Additional file 5: Figure S5.**

**Additional file 6: Table S1.** Linear Regression Analysis for Meta-pool from initial sequencing.

**Additional file 7: Table S2.** Linear Regression Analysis for Replicates combining sequencing lanes.

## Data Availability

The ChIP-seq datasets generated in the current study are available in the NCBI GEO repository, available under GEO accession GSE197960, https://www.ncbi.nlm.nih.gov/geo/query/acc.cgi?acc=GSE197960 *Peaksat* is implemented and freely available under MIT license at https://github.com/FrietzeLabUVM/peaksat
