# Supplementary material for: peaksat: an R package for ChIP-seq peak saturation analysis
Source: BMC Genomics. 2023 Jan 25;24:43. doi: 10.1186/s12864-023-09109-7 (PMC9878872; doi:10.1186/s12864-023-09109-7)
Supplement: Supplementary file 4 — Additional file 4: Figure S4. QC analysis of H4K8ac combined sequencing. [file 12864_2023_9109_MOESM4_ESM.pdf]

S Figure 4

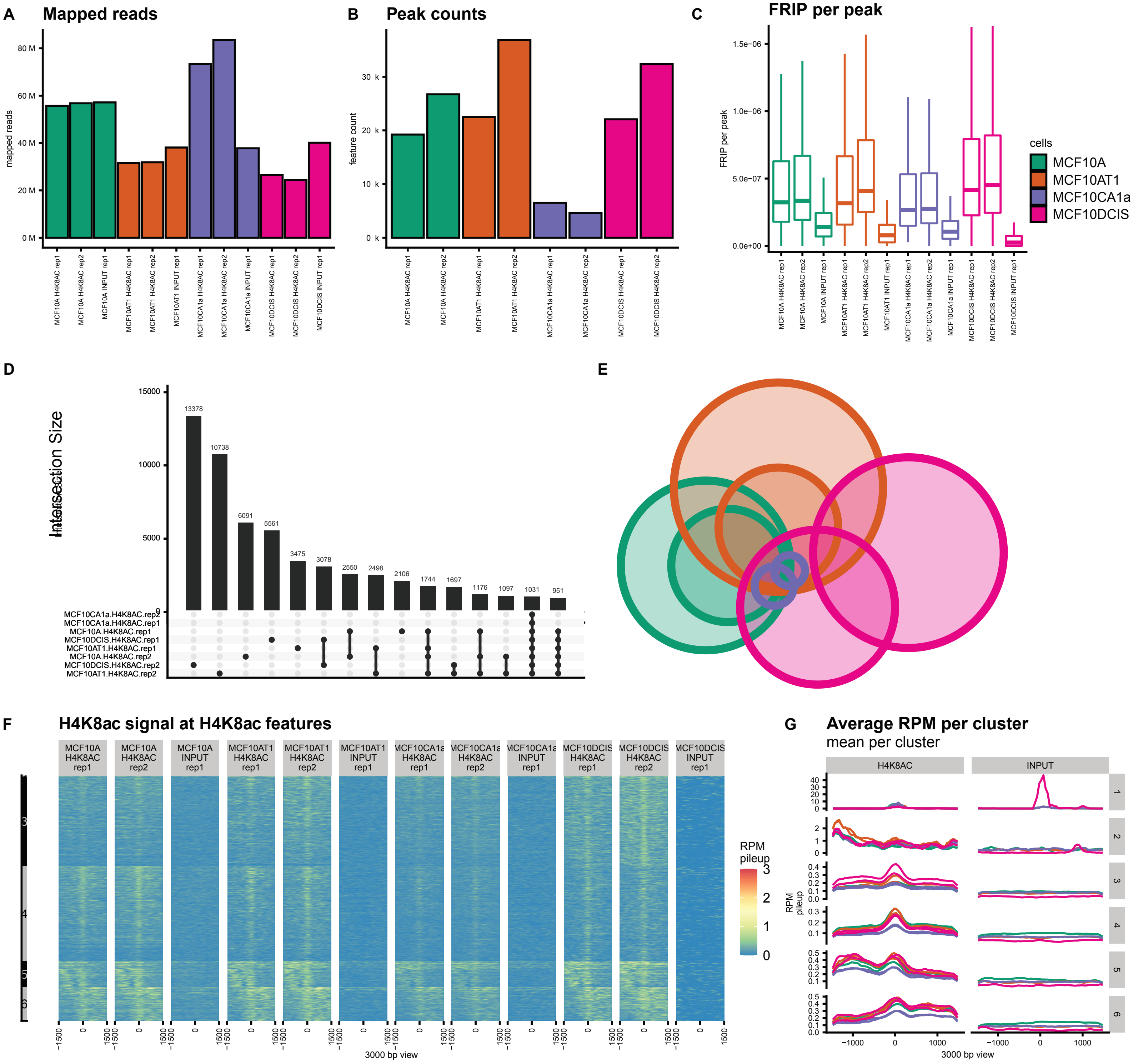

Figure S4: QC analysis of H4K8ac combined sequencing. A) Mapped reads per replicate. **B)** Numbers of peaks called per replicated with a fold-enrichment cutoff of 5. **C)** Boxplots showing the distribution of the fraction of reads in each peak (FRIP per peak) for a common reference peak set of 1000 peaks across each sample. **D)** UpSet plot indicating the commonality and uniqueness of peaks across samples. **E)** Euler plot showing the approximate proportional size and amount of overlap between peak sets. **F)** K-means clustered heatmap of RPM normalized read pileups of 3kb views at the 1000 peak reference set. **G)** Averaged RPM pileups for the clusters indicated in F.
