# Supplementary figures and images for: peaksat: an R package for ChIP-seq peak saturation analysis
Source: BMC Genomics. 2023 Jan 25;24:43. doi: 10.1186/s12864-023-09109-7 (PMC9878872; doi:10.1186/s12864-023-09109-7)

**A**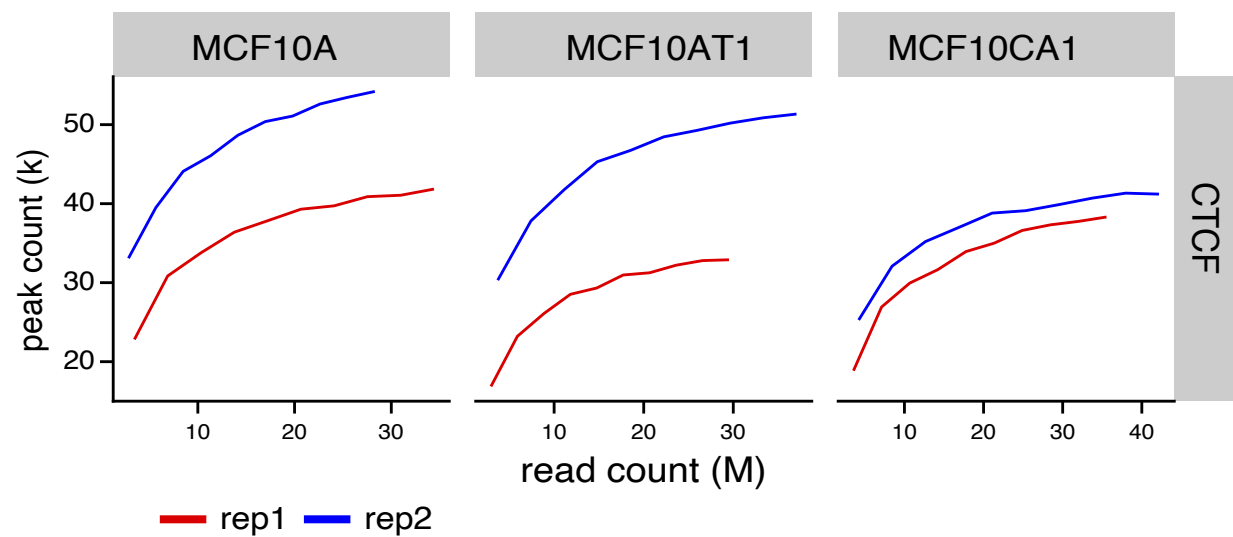**B**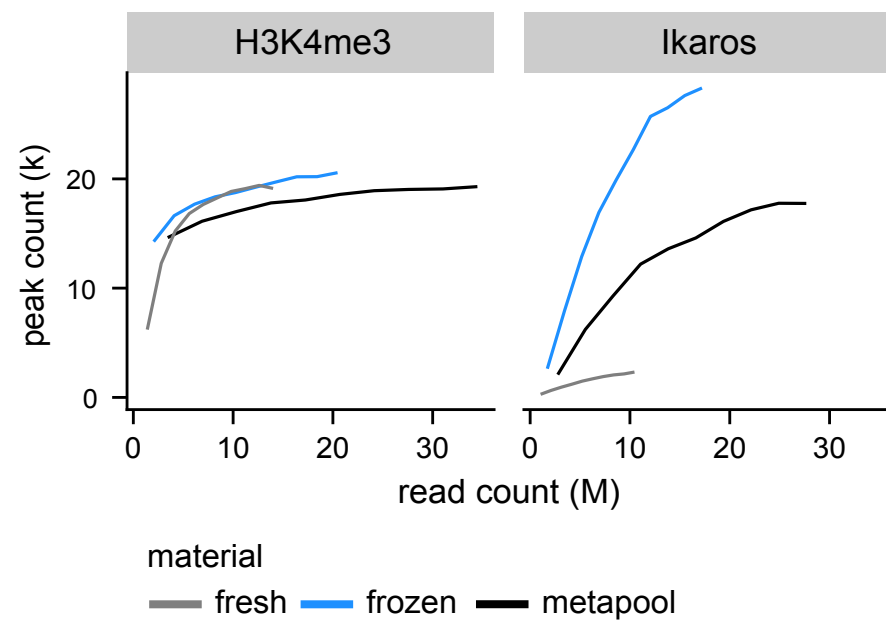**C**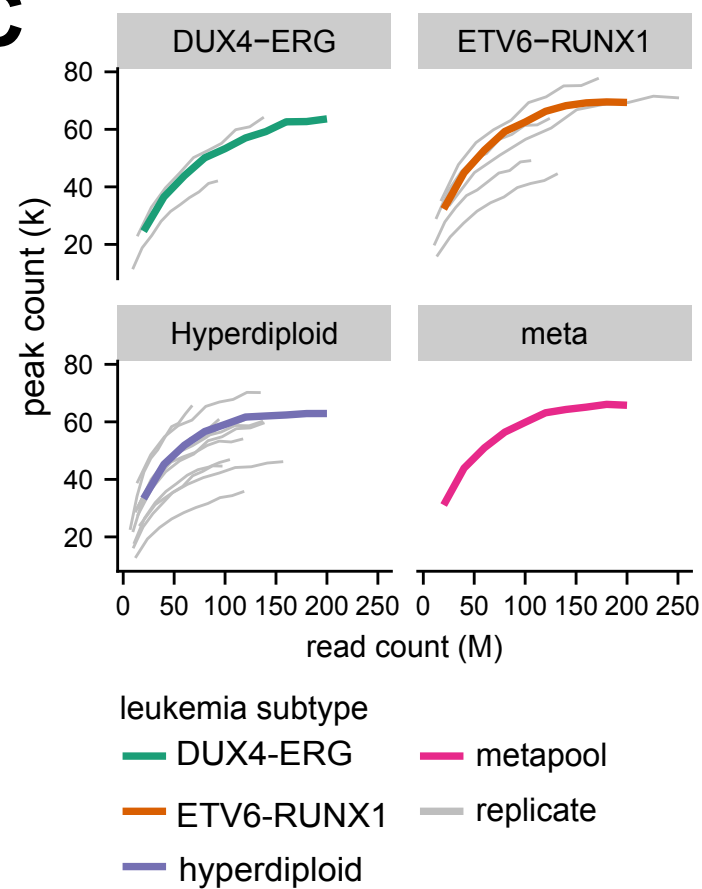

Supplement: Supplementary file 5 — Additional file 5: Figure S5. [file 12864_2023_9109_MOESM5_ESM.pdf]
