# Supplementary material for: peaksat: an R package for ChIP-seq peak saturation analysis
Source: BMC Genomics. 2023 Jan 25;24:43. doi: 10.1186/s12864-023-09109-7 (PMC9878872; doi:10.1186/s12864-023-09109-7)
Supplement: Supplementary file 6 — Additional file 6: Table S1. Linear Regression Analysis for Meta-pool from initial sequencing. [file 12864_2023_9109_MOESM6_ESM.docx]

**Table S1. Linear Regression Analysis for Meta-pool from initial sequencing**

| Mark | H4K5ac | H4K8ac |
| --- | --- | --- |
| Overall Model | R^2^ = 0.8946 | R^2^ = 0.8188 |
| Reduced Model | R^2^ = 0.9972* | R^2^ = 0.9896* |

Overall Model: taking all the 10 down-sampling data points.

Reduced Model: removing the last data points following peak saturation.

R^2^ is collected from the linear regression model and refers to the adjusted R^2^, with all of the p-values fewer than 0.05.
