# Supplementary material for: peaksat: an R package for ChIP-seq peak saturation analysis
Source: BMC Genomics. 2023 Jan 25;24:43. doi: 10.1186/s12864-023-09109-7 (PMC9878872; doi:10.1186/s12864-023-09109-7)
Supplement: Supplementary file 7 — Additional file 7: Table S2. Linear Regression Analysis for Replicates combining sequencing lanes. [file 12864_2023_9109_MOESM7_ESM.docx]

**Table S2. Linear Regression Analysis for Replicates combining sequencing lanes**

| Mark | H4K5ac | | H4K8ac | |
| --- | --- | --- | --- | --- |
|  | rep1 | rep2 | rep1 | rep2 |
| MCF10A | R^2^ = 0.8704^*^ | R^2^ = 0.8164^*^ | R^2^ = 0.8698 | R^2^ = 0.8609 |
| MCF10AT1 | R^2^ = 0.9528 | R^2^ = 0.9775 | R^2^ = 0.919 | R^2^ = 0.8681 |
| MCF10CA1 | R^2^ = 0.9955^*^ | R^2^ = 0.9935^*^ | R^2^ = 0.8243 | R^2^ = 0.8107 |
| MCF10DCIS. | R^2^ = 0.9754 | R^2^ = 0.927 | R^2^ = 0.8791 | R^2^=0.8475 |

For each replicate in each cell line for the specific mark, the left ones are the adjusted R^2^s, then the right side are the p-values of the correspondent linear regression models, with all of the p-values fewer than 0.05. * corresponds to the reduced models removing the outliers after peak saturation.
